# Enhancing relational care through expressions of gratitude: insights from a historical case study of almoner–patient correspondence

**DOI:** 10.1136/medhum-2019-011679

**Published:** 2019-10-04

**Authors:** Giskin Day

**Affiliations:** 1 Florence Nightingale Faculty of Nursing, Midwifery and Palliative Care, King's College London, London, UK; 2 Faculty of Medicine, Imperial College London, London, UK

**Keywords:** cultural history, patient narratives, cultural studies, medical humanities, psychology

## Abstract

This paper considers insights for contemporary medical practice from an archival study of gratitude in letters exchanged between almoners at London’s Brompton Hospital and patients treated at the Hospital’s tuberculosis sanatorium in Frimley. In the era before the National Health Service, almoners were responsible for assessing the entitlement of patients to charitable treatment, but they also took on responsibility for aftercare and advising patients on all aspects of welfare. In addition, a major part of the work of almoners at the Brompton was to record the health and employment status of former sanatorium patients for medical research. Of over 6000 patients treated between 1905 and 1963 that were tracked for the purposes of Medical Research Council cohort studies, fewer than 6% were recorded as ‘lost to follow-up’—a remarkable testimony to the success of the almoners’ strategies for maintaining long-term patient engagement. A longitudinal narrative case study is presented with illustrative examples of types of gratitude extracted from a corpus of over 1500 correspondents’ letters. Patients sent money, gifts and stamps in gratitude for treatment received and for the almoners’ ongoing interest in their welfare. Textual analysis of letters from the almoner shows the semantic strategies that position gratitude as central to the personalisation of an institutional relationship. The Brompton letters are conceptualised as a Maussian gift-exchange ritual, in which communal ties are created, consolidated and extended through the performance of gratitude. This study implicates gratitude as central to the willingness of former patients to continue to engage with the Hospital, sometimes for decades after treatment. Suggestions are offered for how contemporary relational healthcare might be informed by this unique collection of patients’ and almoners’ voices.

## Introduction

Gratitude was described by Solomon in 2004 as ‘one of the most neglected emotions and one of the most underestimated of the virtues’.1 Since then, gratitude has become the subject of a great deal of research especially in the field of positive psychology.2 3 4 This paper considers the insights to be gained from the study of expressions of gratitude in an archive of letters written in the 20th century between almoners at the Brompton Hospital (known now as the Royal Brompton Hospital) and patients who received sanatorium treatment for tuberculosis (TB). The post of ‘hospital almoner’ was initially established in the 1890s to assess patients’ eligibility for charitable medical treatment (‘alms’—hence the title of ‘almoner’).5 Gosling argues that almoners capitalised on the money-handling side of their roles as a route to demonstrating to hospitals the value of their expertise in medicosocial work and so accruing status for the profession.6 7 The importance of the role of the almoner in changing relationships between hospitals and patients is shown by Cullen, who positions the almoner as a skilled intermediary between the hospital and networks of assistance with the aim of attending to the long-term welfare of patients.8 The letters I examine in this study elucidate the complex entanglements of expectation, reciprocation and obligation, where philanthropy was both conferred and received by the almoners and patients. I argue that the performance of gratitude as an intrinsic part of these exchanges helped to consolidate and extend the ties that originated in the communal regimen of the sanatorium.

The first part of the paper considers the ways in which former patients expressed gratitude, both through inscriptions and material gestures. The second part uses a textual analysis of examples of the almoners’ replies to patients to demonstrate the discursive dynamics of reciprocal gratitude. Both approaches highlight how personal, relational exchanges served the transactional, data-collecting exigencies of the communication. I use the lens of Marcel Mauss’ influential 1923–1924 essay *The Gift* 9 to characterise the letters and their contents as constituting a gift-exchange ritual.

Expressions of complaint are dealt with in formal systems in healthcare, but compliments and expressions of gratitude are often overlooked as useful forms of feedback. Yet Herbland *et al* found, in their qualitative analysis, that letters spontaneously sent to an intensive care unit in France were a form of rich, meaningful feedback on quality of care.10 A content analysis of letters of gratitude sent to a palliative care service in Portugal suggests that gratitude could be a quality indicator for care.11 I argue that healthcare organisations could and should do more to acknowledge and enhance gratitude as a key element of relational care. I offer some suggestions for how to improve communicative practices in contemporary healthcare.

## Background

The first almoner at the Brompton Hospital, then known as ‘the Lady Almoner’, was Miss Maurice.12 Her appointment in 1905 coincided with the opening of the Frimley Sanatorium in Surrey for the purpose of rehabilitating selected patients that had been treated for TB in the wards at the Brompton. The Sanatorium’s first Medical Superintendent, Dr Marcus Paterson, devised a scheme of graduated labour, a ‘moral and physical system’ in which patients were given increasingly strenuous tasks starting at grade 1 (‘walking from half a mile to eight miles daily’) and progressing through to grade 6 (‘using a pickaxe, trenching, mixing concrete, felling trees, &c. for six hours daily’).13 Patients were also expected to clean the wards and corridors, polish the brasswork, make their own beds, and generally help to run the Sanatorium. With the appointment of Dr Wilfred Meek as Medical Superintendent in 1912 came the recognition that follow-up records were vitally important to understanding the impact of different treatment regimens. A 1914 report laments the number of patients lost to follow-up (about 30%):14

…it is much to be regretted that so many cases have been lost sight of. This is a difficulty met with in the statistics of all Sanatoria, and is almost inseparable from one like the Brompton Hospital Sanatorium, which draws its patients largely from the working and labouring classes, whose proneness to frequent change of residence is well-known.15

The Medical Research Committee (MRC) in 1917 awarded an annual grant of £150 for the Hospital to investigate the after-histories of patients treated at the Sanatorium.16 This provided the impetus to take follow-up very seriously so that statistically significant data on patients’ progress could be compiled. Meek was succeeded by Dr RC Wingfield as Medical Superintendent in 1919. Wingfield took on the task of compiling decennial reports on the after-histories of patients treated at the Sanatorium. Strenuous efforts were made to trace the 1400 patients with whom contact had been lost. These included circulars of enquiry, advertisements in Sunday papers, letters to the Medical Officers of Health for each district, a search through the death records and a personal canvass at every known address, ‘no stone being left unturned in the endeavour to trace each individual’.17 Over 1000 patients were traced through these methods, leaving only 10% of the 3400 patients treated between 1906 and 1918 as reluctantly having to be recorded as ‘lost sight of’.

The appointment of Miss Lily Constance Marx as Lady Almoner in 1920 brought order to what had been a chaotic system of record keeping. Determined to keep on top of follow-up, Miss Marx would meet, whenever possible, with patients before their discharge from Frimley to stress the importance of keeping in touch for the purposes of research. Enquiries were made every year by writing to, telephoning or visiting every former patient, tracking follow-up appointments at the hospital or collecting intelligence through the dispensaries. Detailed records were kept in a series of case books, organised by year of discharge. In addition to the case books, carbon copies were kept of outgoing correspondence and then matched with patients’ replies. The tenacity of Miss Marx and the Frimley enquiry clerk, Miss W Simkins, paid off. By 1957, of the 6146 former Frimley patients known to still be living in 1946, only 324 had been ‘lost sight of’ and most of those were from before Miss Marx’s appointment. The records clerk, Miss Colgate, estimated in 1957 that fewer than 1% of the 3000 patients admitted since 1946 had been lost to follow-up.18 It was not until 1961 that ‘100% coverage’ for following up former patients ceased to be the primary aspiration of Frimley almoners.19

In spite of a remark from the authors of the MRC Report that ‘with the patients drawn from the [labouring] classes treated at this sanatorium…letter-writing is no congenial task’,20 letters from over 1500 former patients have survived. Along with the information needed for medical research, and the expressions of gratitude that are the focus of this paper, the letters form a unique collection of patient voices not usually heard in the history of medicine. Roy Porter noted in the 1980s that ‘we remain so profoundly ignorant of how ordinary people in the past have actually regarded health and sickness, and managed their encounters with medical men’.21 Although work since has highlighted the historiographic possibilities offered by patient records in medical history,22 23 these records tend to offer little access to the free-form, patient-authored narratives that abound in the Brompton correspondence. The ‘ordinary’ people treated at Frimley prove themselves to be extraordinary through their correspondence with the almoners. The letters are redolent with stories—stories of managing the adversities of war, of stigma from neighbours and sometimes family members, and of health setbacks that accompanied TB in the era before antibiotic treatment.

## Methods of analysis

The value of studying primary texts in the form of letters to understand the history of healthcare has been elaborated by Howell, Rafferty and Snaith, who argue that nurses’ writings are sites of claims for medical, cultural and narrative authority that illuminate current nursing practice.24 The archive of almoners’ correspondence with former Frimley patients spans 1920–1963 and is held on deposit at the Royal London Hospital Archives. It comprises a mix of letters, forms, receipts, informal notes and occasional photographs. The collection is far from complete. The case books refer to letters which are no longer in the files, and there are clear gaps in the correspondence. Generally, carbon copies of typewritten letters from the almoners are interspersed with mostly handwritten patients’ replies. Given the fragmentary nature of the archival holdings, a formal quantitative content analysis would not make for a robust analysis. Instead, certain themes that are apparent within the correspondence are summarised here, with selective use of illustrative extracts that are exemplary for these themes. This approach is endorsed by Riha, who argues that detailed work on selected examples is perhaps more reliable than statistics at reconstructing everyday and, especially, medical life.25

I worked my way twice through the letters from 1506 correspondents over several weeks in successive years noting specific expressions of gratitude. During the first round, the name of the correspondent was entered into a spreadsheet along with brief remarks on the nature of the letters in respect to gratitude. When the archivist kindly granted permission to photograph letters of particular interest, a visual database was compiled using the photo-editing software Picasa. The letters were then tagged with the name of the patient to whom they referred, the year in which they were written, and any additional keywords that referred to the ways in which gratitude was expressed, for example, ‘donation’ and ‘stamps’, and for what patients expressed gratitude, for example, ‘treatment’, ‘enquiries’ and ‘advice’.

## Findings

### Gratitude for treatment

Many former Sanatorium patients expressed gratitude to the institution they considered instrumental in restoring their health at a time when TB was often still deadly. To give a few of many examples:

I have enjoyed splendid health since taking your wonderfull [sic] treatment and must thank you always for it. I remain yours gratefully…26

Thank all at Brompton and Frimley for the kind help and treatment while I was there, and wish many others may be so well. Thanking you all.27

I often think what Brompton Hospital done for me also staff and my Subscriber’s letter.28 I must now thank one and all for restoring me to such fine health, going through last war and still A1 though crippled and wounded with stiff left leg since 1918…. I will now close and it gives me the greatest of pleasure to write these few lines on my health. Thanking once again Subscriber and Brompton Hospital for my health today.29

Along with being grateful for the treatment they received, there is also a sense of pride throughout the correspondence through former patients emphasising that they are keeping up the ‘lessons learned’ at Frimley. When asked to give an account of the work they were doing, if any, many stressed that they were keeping up Frimley habits by exercising out of doors:

I can do light duties and able to go [for] long walks when suitable weather…. After the good time I spent at Frimley I feel I know how to live to keep in good health.30

I think I have kept fit, because I was taught how to live at Frimley, and will always be very grateful for the care and attention I received there.31

It is also noticeable throughout the correspondence that expressions of gratitude are often mentioned in close proximity to mentions of ‘kindness’.

I am very grateful for all the kindness shown me by Dr Wingfield and sisters and nurses in the Sanatorium. Would you please convey my gratitude to them all and accept some for yourself, dear Madam. / Believe me still / Yours Gratefully…32

The extension of gratitude for treatment to whichever almoner was currently in post, as shown in the extract above, is typical for patients who were pleased to still be remembered. Appreciation was often recorded in the case books as ‘grateful letter from pt’, sometimes with the phrases of gratitude transcribed. The almoner also earned patients’ gratitude by being quick to offer advice if patients did report health setbacks. She arranged for patients with respiratory problems to be seen at Brompton, and generally did her best to direct patients to sources of help regardless of the nature of their ailments.

### Relational gratitude

Along with thanks for treatment, correspondents most often expressed gratitude that they were still of interest to the Sanatorium.

I can assure you how much I appreciate your annual letters and is indeed a pleasure to me to give you all the information I can about myself and I have had a wonderful year.33

I should like to say how much I appreciate your keeping in touch with me after so long [26 years since discharge].34

I cherish grateful memories of the kind and wonderful treatment I received both at Brompton and Frimley, now 42 years ago. I thank you very much for your letter and it is nice to realise that you still have such kind interest in my welfare after so many years. Wishing you every success. / Believe me to remain ever yours gratefully / [sig.] / PS Will you please accept the enclosed £1 note as a small contribution to your gratitude box.35

Cherry notes that almoners were ‘often resented’,36 and Doyle says the almoner was often portrayed as ‘a heartless harridan’,37 but in the Brompton correspondence there is a marked absence of rancour aimed at the almoners. Indeed, one correspondent who abhorred the treatment regimen and considered Frimley to be ‘a blot on the medical escutcheon’ said, ‘My most pleasant memory is of my interview with yourself’.38 While the almoner herself was hardly ever the focus of ire, not everyone was pleased to hear from her. There are a handful of letters that request the almoner to exercise discretion because of the stigma that being a patient with TB still engendered, including the fear of ostracisation and even blackmail:

I am now married and my husband has a big dread of tuberculosis and any reference to my previous illness would cause serious domestic difficulties, in fact, he would leave me. […] also, lodgers in the house or other strangers might see the letters & serious consequences may result. I am not ungrateful, but I earnestly request that you do not send any more inquiries.39

I cannot help suggesting that the information sought for mainly statistical reasons should be obtained in person as correspondence is not always confidential and may cause serious trouble and inconvenience for when things are going okay we do not wish some people to know, whose tongues can put us to a disadvantage financially and socially.40

You must understand I am now married & should not like my wife to know of my stay at Frimley. There is nothing to be ashamed of I know, but it is just that feeling everybody should have in my position, so I hope you will not think too badly of me for not disclosing my address. […] Thanking you once again for the good you have done me in the past, also for your kind enquiries.41

These letters reveal the complexities of patients needing to negotiate their commitments to the almoner, fuelled in part by a sense of obligation set up by gratitude, and the fear of the implications of revealing to others that they once had TB. For this reason, the almoner often obtained health reports from relatives or friends of patients, or trusted patients to write of their own volition rather than being sent a reminder. Patients’ preferences for how correspondence should be handled were noted in the case books and underlined in red. It is perhaps the reliance on these case books, in which careful note was made of patients who asked for correspondence to be handled sensitively, that led Bryder to write, in her book on the social history of TB, that the Brompton almoner’s ‘extreme diligence was clearly unappreciated’.42 The letters themselves, however, are full of appreciation, even when patients were circumspect about the nature and value of her enquiries.

### Donations of money, gifts and stamps

One of the most conspicuous ways in which patients expressed gratitude was through sending donations by postal order or cheque along with their letters to the almoner. The almoner always acknowledged donations with gratitude and sent a receipt. An example is an exchange with Mrs EFC, discharged from Frimley in 1910 but still sending annual reports of her health 30 years later. She typically sent 2 shillings and 6 pence (equivalent to about £5 today) with her letters, explaining, for example, it ‘may help in a small way for something sadly needed, it is from my sister and myself’.43 The almoner replied, ‘It is very kind of you and your sister to send a donation…. Your gift is much appreciated’.44 The last letter on file is from 1953 when the contribution sent has almost doubled to 5 shillings.

Some amounts donated by former patients of the Sanatorium were substantial. Mr AdSL, writing from Australia, gave a donation of £25 in 1928 (equivalent to about £1000 today). He asked the almoner for advice on what was needed, and the reply came back that the money could best be spent on comfortable chairs for the women’s recreation room at Frimley. He asked for his generosity to be recorded: ‘Could a small plate be placed on the back of one of the chairs indicating the purport of the gift, say, “from a one time male patient who had benefited by treatment here, with initials A.L.”?’45

Other gifts were also forthcoming. A grateful patient donated books to the Sanatorium library in 1951. A former patient gave a television to Frimley staff in 1956 and also offered the indefinite loan of one to any bedridden patient. A patient, writing 30 years after he was discharged in 1922, sent the almoner books of poetry along with a cheque ‘for cigarettes – not for Ministry’.46 The almoner replied:

Your donation is greatly appreciated and it has been placed in our fund from which we help patients in financial need – not in the Ministry funds! I am not quite sure about its use for cigarettes – they are not always permitted, but we have many patients with financial problems and it will be helpful for one of these.47

Some gifts were sent expressly to the almoner. One of Frimley’s earliest patients, Mr AGB (discharged in 1910), was a steadfast correspondent from New Zealand. He sent a calendar for the almoners’ office every year for 45 years until his death in 1955. His wife wrote:

As he was so ill in the Brompton Hospital in 1910 we were very thankfull [sic] that he lived to the age of 78. I am continuing to send the calendars as he did as a mark of appreciation of the Hospital’s care.48

Mr LB sent a booklet with pictures of Australian scenery some 46 years after he was discharged, and the almoner replied with a photograph of Frimley: ‘I hope that it serves to remind you of old times’.49

When the almoner requested news of the health of former patients during the Second World War in 1943, it came with a plea:

As the high rate of postage is likely to continue, I should be glad if you would kindly try and remember to send me your report next year by 1st February 1944, as this would be a help to us and save the hospital considerable expenditure.50

This correspondent was one of several patients who, with her health report, sent stamps to help the almoner with her work. The stamps were often accompanied by expressions of gratitude:

I am enclosing 2/6 in stamps wishing I could help more, again thanking Brompton and Frimley for all past benefits. Yours gratefully*…* 51

Donations were sometimes wryly seen as compensating for tardiness:

I enclose a book of stamps to make up for people like myself who do not forward their returns until they are applied for, and so cost the hospital stamps. Please don’t waste a stamp by acknowledging this.52

I am sorry I omitted to write to you earlier as requested. The matter was in fact borne in mind but I expect the necessity to “dig for victory” obsessed my mind. For which omission I fine myself ten shillings enclosed as a contribution to Hospital Funds.53

In 1947, the almoner sent some gifts of her own. There are some letters of thanks from patients for the ‘Christmas gift’: food parcels made up of donations from the Commonwealth as part of postwar relief efforts.

### Entitlement and obligation

It would be naïve to assume that the gratitude so evident in the Brompton correspondence signifies an altruistic culture, unmotivated by any sense of obligation. The imperative to cooperate with the collection of health reports was made clear to patients, usually on discharge: ‘You will remember that when leaving the Frimley Sanatorium all patients are asked to keep in touch by sending a report once a year, to say how they are and if they are able to carry on their usual occupation’.54

Thomson has characterised the role of the almoner as poised between support and surveillance,55 which is consistent with Frimley patients being expected to comply with enquiries, often for the rest of their lives. When patients did not respond to the almoner’s enquiries, reminders were sympathetic but quite stern:

I am disappointed to have had no reply from you to my letters in recent years, asking for news of your health, and do hope that your failure to answer does not mean that you are ill and unable to write.…As you know, these reports of yours are of great value to our doctors in their research work, helping them to decide on the most successful and lasting forms of treatment. Their findings are applied for the benefit of our many new patients, and the reports of patients who keep well, are naturally of especial interest. I do hope therefore, that you will continue to cooperate with us in this work by sending me annual news of your health. You can feel that by doing so, you are making a very real contribution to the relief of suffering. I enclose a form for completion, also a stamped addressed envelope for your reply, and I do hope to hear from you soon.56

The moralising tone of this letter was consistent with the regimen at Frimley which was explicitly moral as well as physical.57 Good sanatorium patients were, according to Margaret Coltart, the Head Almoner from 1942, expected to show ‘self-discipline, and the particular brand of unselfishness needed in a community of ill people’, using the opportunity ‘to think and learn about human nature in themselves and other people, as well as how to look after their health’.58

Gratitude as a moral imperative dated back to the Hospital’s Standing Rules laid down in the 1840s in which patients were instructed to ‘return humble and devout thanks to almighty God, at their usual place of worship, for any relief or alleviations of suffering they may have received’.59 Also, patients were required to present a note of thanks to the Governor who had recommended them ‘on pain of being excluded from any future benefit from the Hospital’.60

Although the frequency of donations sent with the health reports to the almoner might suggest that one of the motivations for corresponding with patients was for the purposes of fundraising, the almoner was careful never to imply that a monetary contribution was expected from patients. One correspondent, writing in 1945, some 33 years after discharge, says, ‘Christmastime not auspicious for this sort of thing. Money tighter then. Enclosed please find ten shillings for Hospital with best thanks.’61 The almoner replies:

I…would like you to know that when we write to you, it is not for the purpose of obtaining a donation, but purely in order to obtain your health report and I should be very sorry to think that you send money gifts you cannot easily spare. We are, of course, very grateful for financial help, but the majority of our ex-patients simply send us the information for which we ask and that is all we expect of them.62

**Figure 1:**
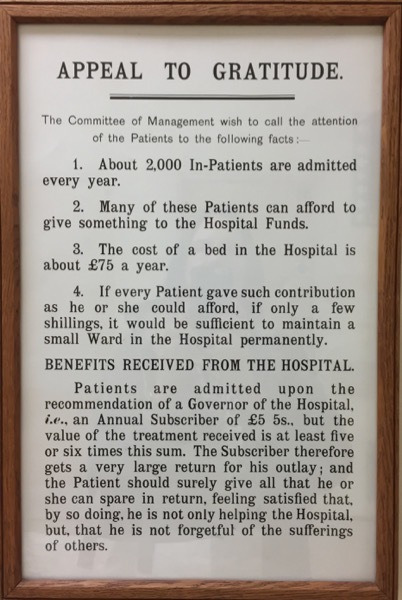
Poster from the Royal Brompton Hospital, c1934.

The insistence that no monetary contributions were expected is consistent with Gosling’s argument that almoners actively tried to counter the widespread impression that their role was merely to handle money.63 But Frimley patients had most likely internalised the message that generosity, no matter how token, was part of the legacy of their treatment under the voluntary hospital system. Figure 1 shows a poster from about 1934, now mounted on the wall in a corridor in the Royal Brompton Hospital as an item of nostalgia but also perhaps as a subtle exhortation to gratitude by current patients. The poster points out the cost of treatment, then paid for by subscriptions from benefactors, but asks patients to give what they could afford, ‘if only a few shillings’. In return, the patient could feel ‘satisfied that, by so doing, he is not only helping the Hospital, but, that he is not forgetful of the sufferings of others’. Most Sanatorium patients passed through the wards of the Hospital before being referred to Frimley, and a sense of financial obligation was almost certainly part of the culture64 in much the same way that charity fundraising efforts are prominent in many of today’s hospitals. The small amounts involved and the ways in which they are framed seem to support Gosling’s contention that money carried meaning for the patient in that it marked their ability ‘to do their bit’.65 These are small but symbolically significant acts of philanthropy, aimed to benefit the almoner’s work, rather than being retrospective payments for treatment.

Acts of 1946 and 1947 establishing the National Health Service (NHS) as free at the point of use caused confusion about whether donations could still be accepted by the Hospital since the state, as one patient put it, was ‘soon to become the Fairy Godmother’.66 The Brompton, however, was recognised in legislation as a ‘teaching hospital’, which meant that, apart from the ownership of the building and equipment which was transferred to the state, the Hospital’s Board of Governors would retain control over the day-to-day running of the Hospital, and there would be ‘no interference with any future gifts to the Hospital, all of which would remain for the Board to spend at their discretion’.67 The ideological dissonance between the state’s discouragement of fundraising and the philanthropic impulse of past patients is encapsulated in a letter from 1952:

Now that you are under the government rule of the thumb, donations I take it don’t interest you now. However I am enclosing p.o. [postal order] for 10/0 to be used as you think best.68

The almoner replies:

I am grateful to you for your help with our research records… Thank you also for your kindness in sending a donation for our funds. Now that the hospital is nationalised we do not, of course, accept gifts towards its upkeep, but help is always welcome for the Almoners’ Fund, from which we help patients with many problems for which funds are not available from official sources.69

The drop-off in contributions after 1948 concerned the almoner who recorded in her January 1953 report that voluntary contributions had more than halved since the previous year. That contributions were considered to be a mix of gratitude and charity is evident:

Although this shows a decrease, it is encouraging to find even this number of patients feeling impelled to send thank-offerings and good wishes for other patients in trouble to get help from the almoners’ department.70

Gradually, the almoner’s role had transitioned from one of giving alms (deeming patients eligible for charitably funded healthcare) to accepting alms from patients. This uncomfortable position—along with the acceptance of paying patients and the shift to local council-funded healthcare and eventually the NHS—led to the term ‘almoner’ becoming anachronistic. In 1949, Miss Coltart proposed that the almoner’s department should be renamed the Social Service Department, because ‘much public ignorance remains as to what is the primary function of almoners’ work’.71 This is consistent with Gosling’s thesis that the introduction of the NHS authorised the reorientation of the profession to legitimise the disentanglement of financial and social work, although the two spheres were never entirely separable.72

Pelletier *et al* argue that linguistic features, such as the giving and giving back of thanks, frame communicative rituals in which autonomy and hierarchy are at stake.73 A close reading of exemplars of the almoners’ correspondence shows how semantic characteristics actively performed gratitude as a means of driving the ongoing gift relationship.

### The almoners’ voice

**Figure 2:**
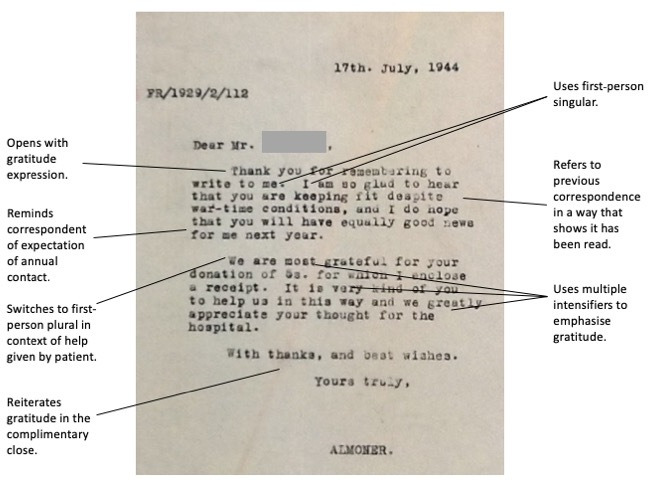
Example of a typical letter from the almoner to a former patient.

From the 1920s through to the late 1950s, the authorial voice in the almoners’ correspondence is remarkably consistent. To read the letters is to imagine that they have been written by one person, familiar with each patient’s history and life circumstances. Figure 2 shows the construction of a typical almoner’s letter, showing how integral gratitude was to the almoners’ interactions with patients. The almoner makes frequent use of intensifying particles, often used at the expense of being concise (eg, thank you *very much*, *most* grateful). Jautz, in a study of thanking routines, finds that intensifiers, along with explicitly stating why one is grateful, lift ‘a mere token of appreciation to a situation-specific expression of one’s personal gratitude’.74 The almoner’s use of first-person singular (‘I’, ‘my’, ‘me’) also helped to personalise what was essentially an institutional relationship.

The job title below the signature changes depending on the year of writing: Lady Almoner (up to 1942), Almoner (1942–1948), Acting Almoner (1948–1949), Frimley Almoner (1950–1959), and ‘Frimley Follow-up Department’ or ‘Follow-up Department “R”’ (1959–1963). This is the only clue, from a patient’s perspective, to a changing cast of record keepers, and it belies the concerted effort by many hands to maintain the work of follow-up alongside other duties. By the time Miss MS Coltart was senior almoner in the late 1940s, six other almoners were employed at the Brompton in various capacities along with a number of record clerks and typists.

One of the first signs of bureaucratic expediency came in 1939, when a form was sent with the almoner’s letter for former patients to fill out their details. The form asked for details of name and address (including a second address ‘which will always find in case of removal’), weight, if having a cough and whether any sputum, details of whether working or not and in what capacity, any special treatment, other information, doctor’s address, and insurer and insurance number if insured (for pre-NHS forms). The form was redesigned in 1940 to omit the request for insurance information and to place the request for any other information after the doctor’s address, allowing extra space for more information that might be helpful.

The form accompanied rather than replaced the almoner’s letter: the personal touch was still very much in evidence. Even so, the forms met with a mixed response. Most respondents seemed happy enough to complete the form, often adding chatty remarks in the space allowed for ‘Any other information you think might be helpful’. A few took exception. One of the first ex-patients to receive the form, having previously cheerfully responded to the almoner’s enquiries, now wrote: ‘I wish you to know I am an Englishman true born, & of good report, and refuse to acknowledge your right to enquire into my private affairs’. He still ended his letter with ‘With every good wish to the medical and nursing staff in their noble work’, before signing off with ‘Believe me, / Yours grossly insulted…’.75 Mr FF, writing in 1944, also took exception to the form, deeming it a ‘waste of time and supplies’.76 The almoner writes back: ‘I am very sorry if the fact you were sent a Frimley record form displeases you; but it is customary to send these forms to our ex-patients, and many of them prefer to fill in a form rather than write a personal note, or telephone or call at the hospital as you usually do. I will see to it that in future no form is sent with our usual enquiry’.77 And she did.

A letter dated 17 July 1957 from the almoner’s clerk, Kathleen Colgate, to the then Medical Superintendent of the Sanatorium reveals the administrative burden engendered by follow-up work.78 Since the previous 10-year block of statistics compiled in 1946, some 3000 extra patients had been added to the records, many of whom changed addresses in the 2-year interval between discharge and follow-up letters being sent out. The improvement in survival of new patients owing to chemotherapy meant that most patients were now available for 5-year follow-up, and the technical information required was much more demanding than merely the determining of whether patients were alive and able to work. The Almoner’s Report Book kept by Miss Coltart catalogues ongoing problems with retaining clerical staff and a burgeoning workload.79 The decision was made to step down follow-up work.

In 1958, letters from the almoner to earlier patients became a gentle ‘thank-you and goodbye’. Mr JS, a Frimley patient in 1909 whose correspondence over nearly five decades is filled with gratitude, was informed:

As modern methods of treatment have revolutionised the field of chest illnesses, we are no longer following up our earlier patients, but I shall always be pleased to hear from you and to see you if you come to London.… Many thanks for all the help that you have given by reporting for so many years for our research.80

**Figure 3:**
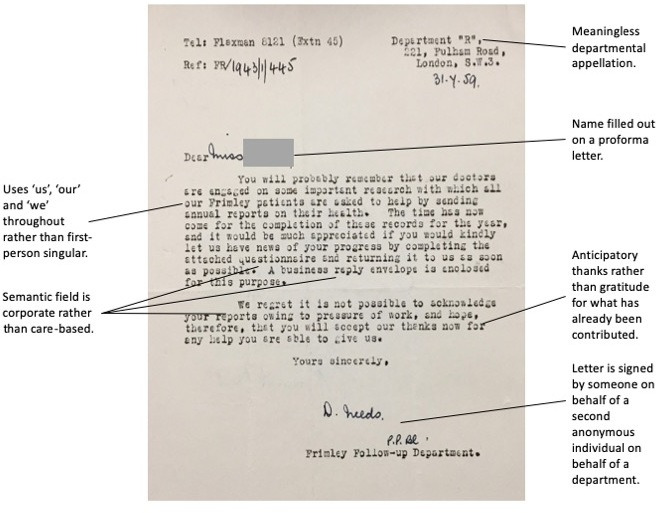
Example of a typical letter from ‘Frimley Follow-up Department’ to a former patient.

More recent patients were still asked for reports of their health in 1959, but the letters had now begun to take on a corporate register, form and feel (Figure 3).

A key difference between the requests for reports from 1944 (Figure 2) compared with 1959 (Figure 3) is the semantic switch from pleasure (glad, fit, hope, good, grateful, kind, help, appreciate) to one of encumbrance (‘time has come’, completion, records, questionnaire, regret). Whereas Miss Marx in her report in 1920 had written that ‘the very considerable time and labour expended is more than compensated for by [patients’] gratitude’,81 the burden of collecting data had come to outweigh their usefulness and no amount of gratitude from patients could compensate for this. Follow-up was discontinued in 1960.

Nostalgia for the voluntary hospitals and scepticism about the NHS were hard to shake. A retired nurse, treated at Frimley in 1925, writes bitterly in 1960:

I suppose I am fortunate to be here, but it is hard going. There is no mercy in the National Health Service. It was the worst thing that the late Aneurin Bevan did to kill the Voluntary Hospitals as such. Up here in Lancashire things are very different from London and the South. The people are hard and very callous.82

Gratitude as a frequently evoked emotion associated with the NHS83 was still some way off for those who had benefited from the voluntary hospital system.

## Strengths and limitations

A particular strength of this study is that it draws on a longitudinal sample of letters in which, independently of each other, multiple patients repeatedly and robustly expressed gratitude. Having both sides of the correspondence, and a full account of the circumstances in which it was produced, is unusual in epistolary research. It enables the exploration of the dynamics of call-and-response in the production and reaffirmation of institutionally proscribed relationships.

A limitation is that gratitude is undoubtedly over-represented in the correspondence, given that patients were alive at the time of writing and therefore more likely to implicate sanatorium treatment for their well-being. Also, only patients who had been at Frimley Sanatorium for more than 28 days were included in follow-up, meaning that voices are not included of those who discharged themselves, presumably dissatisfied with treatment at Frimley (although apparently this rarely happened according to Dr Wingfield’s obituarist).84 Patients were required to submit completely to a strictly timetabled regimen at the Sanatorium85: there was no room for dissent. Patients’ willingness to comply with authority may well have contributed to the high response rates to the almoners’ enquiries.

What cannot be adequately conveyed in an analysis of this nature is the materiality of the original letters: the feel and weight of the paper (poorer quality during war years), the use of different writing implements, and the effort taken with handwriting—especially evident with increasing frailty of elderly former patients. We can only speculate on the influence of these intangible features, but it underlines the value to historians of emotions of engaging with primary sources rather than digital renditions in seeking to apprehend the affect generated by letters.

## Discussion and conclusion

Far from pursuing a narrow moral agenda or being a managerialist ploy to elicit greater productivity, gratitude—sincerely expressed at the interpersonal level—contributed to the durability of relationships in this case study of correspondence between staff and patients. Although the correspondence was primarily intended to collect data for statistical purposes, the quantitative outputs in the form of reports of the after-histories of patients13 86 87 have nothing to say to the rich, lived experiences that unfold through the pages of letters. It is through engagement with the hallmarks of gratitude in the correspondence that one gains an insight into the affirming, emotional heft of the work that offsets the administrative burden of needing to keep track of thousands of patients.

My interest in gratitude is, in part, motivated by concerns over morale in the NHS. The King’s Fund has highlighted low morale as a significant problem in the NHS, with a major contributing factor being that staff feel undervalued.88 The Royal College of Nursing Employment Survey 2017 found that the majority of nursing staff felt overworked, underpaid and unable to provide a satisfactory level of patient care, with over a third planning to leave the profession.89 A survey of 929 general practitioners in the South of England in 2017 found that 59.4% reported that morale had reduced over the past 2 years, and 48.5% said they had brought forward their plans to leave general practice.90 Despite this, patient feedback consistently shows high levels of satisfaction with the care delivered by doctors and nurses in the NHS.91 92 The relentless emphasis on what needs improvement in healthcare risks disproportionately emphasising negative phenomena. This is at the expense of learning from heliotropic factors—those positive conditions that contribute to human flourishing.93

There is a fair amount of cynicism attached to scholarship that focuses on the ‘positive’. It implies a preconceived bias which is contrary to the value-neutral position usually seen as the hallmark of scientific enquiry. Ironically, the word ‘critical’ attracts no such censure. ‘Positive thinking’ has been characterised as delusional and destructive,94 and emblematic of the corporatisation of emotion.95 Care Opinion, a website where people can share experiences of healthcare, recently was described as ‘close to pointless as it mostly provides a channel for praise’.96 The implication is that it is only complaints that drive change. However, this notion is challenged by an analysis of 41 discursive interviews with NHS staff in which interviewees mentioned ‘patient and family complaints’ as events that had significantly negative effects on staff.97 Participants described the emotional impact of complaints, for example, ‘gutting’, ‘devastation’, ‘awful shame’, ‘disbelief’, ‘shock’ or ‘incomprehension’, but hardly ever described complaints raised by patients as grounds for improving the quality of care. Alexander has pointed out that positive stories are an effective way to share good practice and support interprofessional learning.98 She links positive feedback to having a powerful impact in lifting staff morale: ‘Online feedback is not just data: it is often an intervention, an act of encouragement, support and solidarity with public service staff’.

Pelletier *et al* recently demonstrated the value of using Marcel Mauss’ concept of the gift in analysing exchanges in medicine that have ritualistic and performative aspects.99 Mauss elaborated the idea that gifts are not disinterested. They participate in economies of gift exchange in communities, and the expectation of reciprocity consolidates social ties. In the Brompton correspondence, patients’ gifts took the form of material goods—donations, stamps, presents and so on—but the most valued gift was the information that enabled the almoner to participate in other circles of gift exchange within the knowledge community of the hospital: the gifting of data to the doctors compiling research on the after-histories of patients with TB. This concept of knowledge exchange is in keeping with work by Konstantinou and Fincham which shows how gift relations of reciprocation and obligation enhance working capability.100 However, there is more at stake in the Brompton case study than the gift relationship merely enabling the work of the almoners. The correspondence took the form of an annual ritual that *performed* the continuation of care. It exemplifies what Mauss describes as ‘the solicitude arising from reciprocity and co-operation’.101

What, then, can we learn from the Brompton correspondence about the role of gratitude in relationship building in contemporary healthcare?

1. Gratitude requires opportunities for expression. The exigency of gathering data from former sanatorium patients for research purposes had the happy side effect of giving patients the chance to express gratitude and the almoners the chance to reciprocate. That the gratitude was unsolicited, rather than part of a formal service-oriented feedback process, made it come across as an unforced gift—not without the obligation to reciprocate, but also not purely instrumentalist.
2. The Brompton correspondence shows that, regardless of who was writing the letters, a familiarity with previous correspondence and the semantic strategy of using the first person singular (‘I’, ‘me’ and ‘my’) created continuity in a way that transcended politeness and made patients feel acknowledged and valued. This is one of the ways in which care can become more personal, as called for the in the NHS Long-Term Plan.102
3. Contemporary strategies for effective communication with patients could be strengthened if healthcare professionals demonstrate familiarity with information previously disclosed by a patient. Boilerplate correspondence—the use of standard letter templates—has its advantages in disseminating information, but it is the antithesis of patient-centredness. Millions of letters are written and received within the NHS each month.103 Correspondence that is devoid of personalisation, bar name and address, may reinforce patients’ perceptions that they are part of a workflow process rather than of interest as an individual to caregivers.
4. Complaints and compliments are not mutually exclusive. Patients who complained about follow-up were keen to stress that they were not ungrateful. A simplistic dichotomy of counting complaints versus compliments, often implemented in healthcare feedback mechanisms, does not do justice to the nuances of patient feedback. Although patient satisfaction is meant to be a measure of whether expectations have been met, studies show that there is considerable variation at the patient level where satisfaction depends on a range of factors extraneous to a consultation.104 105 Patient satisfaction measures are particularly poor for yielding sensible data because patients are often asked about factors that are outside of the control of the person for whom they are providing feedback.106 It is to be hoped that the recent review announced by NHS England of the much-maligned Friends and Family Test107 will consider the ways in which gratitudinous feedback is captured, acknowledged, recorded and directed.

Did the expression and reception of gratitude in the Brompton correspondence enhance the subjective well-being of the patients and staff? We cannot know for sure, but the material and linguistic markers of pleasure certainly point in this direction. There is a considerable body of evidence that expressing gratitude increases well-being. A scoping review of literature on gratitude expressed to health professionals found that gratitude is likely to have personal and professional impacts, with potential to affect motivation and retention.108 A review of gratitude research carried out by Wood, Froh and Geraghty shows that multiple studies repeatedly and robustly correlate gratitude with well-being.109 Davis *et al* carried out a meta-analysis of gratitude interventions such as gratitude lists, journaling and letter writing, finding that they show promise for improving psychological well-being.110 A study found that writing letters of gratitude increased participants’ happiness and life satisfaction, while decreasing depressive symptoms.111 A small randomised control trial of an intervention that involved healthcare practitioners keeping gratitude diaries led to a reduction in perceived stress and depressive symptoms.112 The writing and receiving of letters between the almoners and former patients in which gratitude was the dominant emotion might well have had a similarly positive effect.

It is intended to expand on the work reported here. Future work will involve an ethnographic study with a view to providing guidance on how to better recognise and facilitate the gratitude in contemporary healthcare settings.

### Patient and public involvement

This is an archival study and patients and the public were not involved in the design or implementation.

## Notes

1. Robert C. Solomon (2004), “The Psychology of Gratitude: Foreword,” in *The Psychology of Gratitude*, ed. Robert A. Emmons and Michael E. McCullough (Oxford: Oxford University Press), v.

2. Don E. Davis et al. (2016), “Thankful for the Little Things: A Meta-Analysis of Gratitude Interventions,” *Journal of Counseling Psychology* 63, no. 1: 20–31, doi:10.1037/cou0000107.

3. Christina N. Armenta, Megan M. Fritz, and Sonja Lyubomirsky (2017), “Functions of Positive Emotions: Gratitude as a Motivator of Self-Improvement and Positive Change,” *Emotion Review* 9, no. 3: 183–90, doi:10.1177/1754073916669596.

4. Blaire Morgan, Liz Gulliford, and Kristján Kristjánsson (2017), “A New Approach to Measuring Moral Virtues: The Multi-Component Gratitude Measure,” *Personality and Individual Differences* 107: 179–89, doi:10.1016/j.paid.2016.11.044.

5. George Campbell Gosling (2018), “Gender, Money and Professional Identity: Medical Social Work and the Coming of the British National Health Service,” *Women’s History Review* 27, no. 2: 312, doi:10.1080/09612025.2017.1328760.

6. George Campbell Gosling (2017), *Payment and Philanthropy in British Healthcare, 1918–48* (Manchester: Manchester University Press).

7. George Campbell Gosling (2018), “Gender, Money and Professional Identity.”

8. Lynsey T. Cullen (2013), “The First Lady Almoner: The Appointment, Position, and Findings of Miss Mary Stewart at the Royal Free Hospital, 1895–99,” *Journal of the History of Medicine and Allied Sciences* 68, no. 4: 551–82, doi: 10.1093/jhmas/jrs020.

9. Marcel Mauss (2000), *The Gift* (New York: W.W. Norton & Company).

10. Alexandre Herbland et al. (2017), “Thank You Letters from Patients in an Intensive Care Unit: From the Expression of Gratitude to an Applied Ethic of Care,” *Intensive and Critical Care Nursing* 43: 47–54, https://doi.org/10.1016/j.iccn.2017.05.007.

11. María Aparicio et al. (2017), “What Are Families Most Grateful for after Receiving Palliative Care? Content Analysis of Written Documents Received: A Chance to Improve the Quality of Care,” *BMC Palliative Care* 16, no. 1: 47, doi:10.1186/s12904-017-0229-5.

12. Margaret Coltart, 1942, *Almoner’s Report Book* (1948–1966).

13. Percival Horton-Smith Hartley, Rodolph Charles Wingfield, and V.A. Burrows (1935), *The Expectation of Survival in Pulmonary Tuberculosis: An Analysis of 8,766 Cases Treated at the Brompton Hospital Sanatorium, Frimley, Brompton Hospital Reports 4.1* (London).

14. For an account of admissions, finances and staffing during the early years of Frimley Sanatorium—including the role of the almoner—refer to P.J Bishop and B.D.B. Lucas (1991), *The Seven Ages of the Brompton: A Saga of a Hospital* (London: The Board of Governors, Royal Brompton National Heart and Lung Hospitals), 87–105.

15. S.H. Haberson et al. (1914), *Report on the After-Histories of Patients Discharged from the Brompton Hospital Sanatorium at Frimley, in Surrey, During the Years 1905–1910: Medical Research Council Special Report Series No. 85* (London: HMSO), 5.

16. Medical Research Committee (1917), *Minutes of Meetings* (London).

17. Percival Horton-Smith Hartley, R.C. Wingfield, and J.H.R. Thompson (1924), *An Inquiry into the After-Histories of Patients Treated at the Brompton Hospital Sanatorium at Frimley, during the Years 1905–1914* (London), 13.

18. Kathleen Colgate to Dr Foster-Carter, March 14, 1957.

19. Margaret Coltart, 1942, *Almoner’s Report Book*, July 9, 1962.

20. Percival Horton-Smith Hartley, R.C. Wingfield, and J.H.R. Thompson (1924), *An Inquiry into the After-Histories of Patients*, 12.

21. Roy Porter (1985), “The Patient’s View: Doing Medical History from Below,” *Theory and Society* 14, no. 2: 175–98, 176.

22. G.B. Risse and J.H. Warner (1992), “Reconstructing Clinical Activities: Patient Records in Medical History,” *Social History of Medicine* 5, no. 2: 183–205, doi: 10.1093/shm/5.2.183.

23. Jonathan Gillis (2006), “The History of the Patient History Since 1850,” *Bulletin of the History of Medicine* 80, no. 3: 490–512, doi: 10.1353/bhm.2006.0097.

24. Jessica Howell, Anne Marie Rafferty, and Anna Snaith (2011), “(Author)Ity Abroad: The Life Writing of Colonial Nurses,” *International Journal of Nursing Studies* 48, no. 9: 1155–62, doi:10.1016/j.ijnurstu.2011.03.010.

25. Ortun Riha, 1995, “Surgical Case Records as an Historical Source: Limits and Perspectives,” *Social History of Medicine* 8, no. 2: 271–83, doi: 10.1093/shm/8.2.271.

26. LF to Almoner, April 10, 1943.

27. BW to Almoner, March 2, 1944.

28. Patients could be admitted on the recommendation of a Subscriber—an individual who contributed annually to the upkeep of the Hospital.

29. FCC to Almoner, March 17, 1942.

30. MAF to Almoner, October 25, 1943.

31. MS to Almoner, July 27, 1954.

32. ARB to Almoner, November 16, 1961.

33. CHC to Almoner, May 13, 1936. Patient was discharged in 1914.

34. IL to Almoner, May 31, 1943.

35. AN to Almoner, April 1952.

36. Steven Cherry (1996), “Accountability, Entitlement, and Control Issues and Voluntary Hospital Funding *c*1860–1939,” *Social History of Medicine* 9, no. 2: 215–33, doi: 10.1093/shm/9.2.215.

37. Barry M. Doyle (2014), *The Politics of Hospital Provision in Early Twentieth-Century Britain* (London: Pickering & Chatto), 69.

38. JR to Almoner, January 8, 1936.

39. KB to Almoner, January 11, 1936.

40. FS to Almoner, n.d.

41. WGK to Almoner, August 31, 1932.

42. Linda Bryder (1988), *Below the Magic Mountain: A Social History of Tuberculosis in Twentieth-Century Britain* (Oxford: Clarendon Press), 220.

43. EFC to Almoner, April, 1940.

44. Almoner to EFC, April 16, 1940.

45. Letter from AdSL to Almoner, December 6, 1928.

46. SG to Almoner, February 18, 1955.

47. Almoner to SG, February 22, 1955.

48. AEB to Almoner, November 9, 1955.

49. LB to Almoner, January 26, 1953.

50. Almoner to EMP, March 24, 1943.

51. MAF to Almoner, October 25, 1943.

52. APM to Almoner, July 3, n.d. (probably 1944).

53. TMHM to Almoner, May 17, 1942.

54. Almoner to GS, February 6, 1935.

55. Elaine Thomson (2002), “Between Separate Spheres: Medical Women, Moral Hygiene and the Edinburgh Hospital for Women and Children,” in *Medicine, Health and the Public Sphere in Britain, 1600–2000*, ed. Steve Sturdy (Abingdon: Routledge), 107–22.

56. Almoner to AMG, January 3, 1948.

57. Percival Horton-Smith Hartley, R.C. Wingfield, and J.H.R. Thompson (1924). *An Inquiry into the After-Histories of Patients*, 12.

58. Margaret Coltart, Helen Raine, and Elizabeth Harrison (1959), *Social Work in Tuberculosis* (London: The Chest and Heart Association).

59. P.J. Bishop and B.D.B. Lucas (1991), *The Seven Ages of the Brompton*, 19.

60. P.J. Bishop and B.D.B. Lucas (1991), 19.

61. GER to Almoner, received January 10, 1946.

62. Almoner to GER, January 17, 1946.

63. George Campbell Gosling (2018), “Gender, Money and Professional Identity”.

64. For a discussion of the contributory arrangements of patients under the voluntary hospital system, see Martin Gorsky, Martin Powell, and John Mohan (2002), “British Voluntary Hospitals and the Public Sphere: Contribution and Participation before the National Health Service,” in *Medicine, Health and the Public Sphere in Britain, 1600–2000,* ed. Steve Sturdy (Abingdon: Routledge), 123–44.

65. George Campbell Gosling (2018), “Gender, Money and Professional Identity”, 323.

66. HRWM to Almoner, January 14, 1946.

67. P.J. Bishop and B.D.B. Lucas (1991), *The Seven Ages of the Brompton*, 156.

68. FD to Almoner, received February 23, 1952.

69. Almoner to FD, February 25, 1952.

70. Coltart, *Almoner’s Report Book*, January 12, 1953.

71. Coltart, *Almoner’s Report Book*, March 8, 1949.

72. George Campbell Gosling (2018), “Gender, Money and Professional Identity”.

73. Caroline Pelletier et al. (2018), “The Gift in A&E: Re-Framing the Medical Case Presentation through Mauss,” *Social Theory & Health* 4: 1–18. Published electronically December 14, doi:10.1057/s41285-018-00086-6.

74. Sabine Jautz (2015), *Thanking Formulae in English: Explorations Across Varieties and Genres* (Amsterdam: John Benjamins Publishing Company), 102.

75. CQ to Almoner, February 28, 1939.

76. FF to Almoner, April 20, 1944.

77. Almoner to FF, April 22, 1944.

78. Kathleen Colgate to Dr Foster-Carter, July 17, 1957.

79. Coltart, *Almoner’s Report Book*.

80. Almoner to JHS, January 29, 1958.

81. Laura Constance Marx (1921), *The Eightieth Annual Report of the Hospital for Consumption and Diseases of the Chest*, Brompton, Read at the Annual Court of Governors, 17th March, 1921 (London), xvii.

82. EMW to Almoner, November 9, 1960.

83. Research Works (2016), *NHS Identity Research Phase One and Two Combined Research Report*, https://www.england.nhs.uk/nhsidentity/wp-content/uploads/sites/38/2016/08/NHS-Identity-Research-phase-one-and-two.pdf.

84. FHY (1946), “In Memoriam: Rodolph Charles Wingfield, BM (Oxon), FRCP (Lond)”, *British Journal of Tuberculosis and Diseases of the Chest* 40, no. 2: 1–4.

85. P.J. Bishop and B.D.B. Lucas (1991), *The Seven Ages of the Brompton*.

86. S.H. Haberson et al. (1914), *Report on the After-Histories of Patients Discharged*.

87. Percival Horton-Smith Hartley, R.C. Wingfield, and J.H.R. Thompson (1924), *An Inquiry into the After-Histories of Patients*.

88. The King’s Fund (2018), “How Is the NHS Performing?”, *March 2018 Quarterly Monitoring Report*, https://www.kingsfund.org.uk/publications/how-nhs-performing-march-2018.

89. Rachel Marangozov et al. (2017), *Royal College of Nursing Employment Survey 2017*, https://www.rcn.org.uk/professional-development/publications/pdf-007076.

90. Katherine Owen et al. (2019), “GP Retention in the UK: A Worsening Crisis. Findings from a Cross-Sectional Survey,” *BMJ Open* 9, no. 2: e026048, doi:10.1136/BMJOPEN-2018-026048.

91. Linda H. Aiken et al. (2018), “Patient Satisfaction with Hospital Care and Nurses in England: An Observational Study”, *BMJ Open* 8, no. 1 (January 11): e019189, doi:10.1136/bmjopen-2017-019189.

92. Care Quality Commission (2018), NHS Patient Survey Programme 2017 Adult Inpatient Survey Statistical Release, https://www.cqc.org.uk/publications/surveys/adult-inpatient-survey-2017.

93. Kim S. Cameron and Gretchen M. Spreitzer (2011), *The Oxford Handbook of Positive Organizational Scholarship* (Oxford University Press, USA).

94. Barbara Ehrenreich (2010), *Smile or Die* (London: Granta).

95. William Davies (2015), *The Happiness Industry: How the Government and Big Business Sold Us Well-Being* (London and New York: Verso).

96. John Wilson (2018), “Practical Measures to Heal the NHS in Scotland,” *Holyrood Magazine*, December, https://www.holyrood.com/articles/comment/practical-measures-heal-nhs-scotland.

97. Mary Adams, Jill Maben, and Glenn Robert (2018), “'It's Sometimes Hard to Tell What Patients Are Playing at’: How Healthcare Professionals Make Sense of Why Patients and Families Complain about Care”, *Health: An Interdisciplinary Journal for the Social Study of Health, Illness and Medicine* 22, no. 6: 603–23, doi:10.1177/1363459317724853.

98. Gina Alexander (2019), “Health Services Learn from Both Positive and Negative Feedback,” *Holyrood Magazine*, January, https://www.holyrood.com/articles/comment/health-services-learn-both-positive-and-negative-feedback.

99. Caroline Pelletier et al. (2018), “The Gift in A&E”.

100. Efrosyni Konstantinou and Robin Fincham (2011), “Not Sharing But Trading: Applying a Maussian Exchange Framework to Knowledge Management”, *Human Relations* 64, no. 6: 823–42, doi:10.1177/0018726710388676.

101. Marcel Mauss (2000), *The Gift*, 69.

102. Harry Quilter-Pinner (2018), “The NHS Long-Term Plan: Lessons from the Lord Darzi Review of Health and Care”, https://www.ippr.org/files/2018-11/long-term-nhs-plan.pdf.

103. AMRC (2018), “Please, Write to Me. Writing Outpatient Clinic Letters to Patients. Guidance”, London, http://www.aomrc.org.uk/reports-guidance/please-write-to-me-writing-outpatient-clinic-letters-to-patients-guidance/.

104. C. Salisbury, M. Wallace, and A. A Montgomery, 2010, “Patients’ Experience and Satisfaction in Primary Care: Secondary Analysis Using Multilevel Modelling”, *BMJ (Clinical Research*) 341: c5004, doi:10.1136/bmj.c5004.

105. Martin J. Roberts et al. (2014), “Understanding High and Low Patient Experience Scores in Primary Care: Analysis of Patients’ Survey Data for General Practices and Individual Doctors”, *British Medical Journal* 349: g6034.

106. Jeannie L. Haggerty (2010), “Are Measures of Patient Satisfaction Hopelessly Flawed?”, *BMJ (Clinical Research*) 341: c4783, doi:10.1136/bmj.c4783.

107. Neil Churchill (2019), “Times and Questions May Be A-Changing”, *NHS England Blog*, https://www.england.nhs.uk/blog/times-and-questions-may-be-a-changing/.

108. Maria Aparicio et al. (2019), “Gratitude between Patients and Their Families and Health Professionals: A Scoping Review”, *Journal of Nursing Management* 27, no. 2: 286–300, doi: 10.1111/jonm.12670.

109. Alex M. Wood, Jeffrey J. Froh, and Adam W. A. Geraghty (2010), “Gratitude and Well-Being: A Review and Theoretical Integration”, *Clinical Psychology Review* 30: 890–905, doi:10.1016/j.cpr.2010.03.005.

110. Don E. Davis et al. (2016), “Thankful for the Little Things”.

111. Steven M. Toepfer, Kelly Cichy, and Patti Peters, 2012, “Letters of Gratitude: Further Evidence for Author Benefits”, *Journal of Happiness Studies* 13, no. 1: 187–201, doi:10.1007/s10902-011-9257-7.

112. Sheung-Tak Cheng, Pui Ki Tsui, and John H.M. Lam (2015), “Improving Mental Health in Health Care Practitioners: Randomized Controlled Trial of a Gratitude Intervention”, *Journal of Consulting and Clinical Psychology* 83, no. 1: 177–86, doi:10.1037/a0037895.
